# Effects of Non-Elevation-Focalized Linear Array Transducer on Ultrasound Plane-Wave Imaging

**DOI:** 10.3390/s16111906

**Published:** 2016-11-12

**Authors:** Congzhi Wang, Yang Xiao, Jingjing Xia, Weibao Qiu, Hairong Zheng

**Affiliations:** Shenzhen Institutes of Advanced Technology, the Chinese Academy of Science, 1068 Xueyuan Avenue, Shenzhen University Town, Xili, Nanshan, Shenzhen 518055, China; cz.wang@siat.ac.cn (C.W.); yang.xiao@siat.ac.cn (Y.X.); jj.xia@siat.ac.cn (J.X.)

**Keywords:** ultrafast ultrasound imaging, acoustic lens, non-elevation-focalized transducer, image contrast

## Abstract

Plane-wave ultrasound imaging (PWUS) has become an important method of ultrasound imaging in recent years as its frame rate has exceeded 10,000 frames per second, allowing ultrasound to be used for two-dimensional shear wave detection and functional brain imaging. However, compared to the traditional focusing and scanning method, PWUS images always suffer from a degradation of lateral resolution and contrast. To improve the image quality of PWUS, many different beamforming algorithms have been proposed and verified. Yet the influence of transducer structure is rarely studied. For this paper, the influence of using an acoustic lens for PWUS was evaluated. Two linear array transducers were fabricated. One was not self-focalized in the elevation direction (non-elevation-focalized transducer, NEFT); the other one was a traditional elevation-focalized transducer (EFT). An initial simulation was conducted to show the influence of elevation focusing. Then the images obtained with NEFT on a standard ultrasound imaging phantom were compared with those obtained with EFT. It was demonstrated that, in a relatively deep region, the contrast of an NEFT image is better than that of an EFT image. These results indicate that a more sophisticated design of ultrasound transducer would further improve the image quality of PWUS.

## 1. Introduction

In conventional ultrasound imaging (US), one 2-D image is acquired by a set of scan lines (usually 128 or 256 scan lines). Each scan line is obtained by beamforming the ultrasound echo signals with the delay-and-sum (DAS) method following a focused beam transmission [1]. Therefore, multiple rounds of ultrasound transmitting and receiving are necessary and the frame rate is intrinsically low (about 30–50 frames per second, fps).

To expedite the frame rate, plane-wave ultrasound imaging (PWUS), where the frame rate can be significantly increased to 10,000–20,000 fps, has been well-studied in recent years. It has been used for shear wave tracking in a real-time elastography, and for flow measurement in large arteries [2,3,4,5,6,7]. In PWUS, all the elements of the linear-array transducer are excited at the same time and a plane-wave emission is formed; then the echo signals are processed by the beamformer algorithms to generate one 2-D image [8,9,10,11]. However, although PWUS can significantly increase the imaging frame rate, the image quality degrades compared to a conventional US image if only the traditional beamformer algorithm is used. To solve this problem, complex algorithms such as coherent compounding [12] or regularization based on compressive sensing frame [6,7,13,14] have been proposed and h studied. Another modification which may be helpful for enhancing the image quality of PWUS, i.e., changing the structure of ultrasound transducer, has rarely been studied.

**Figure 1:**
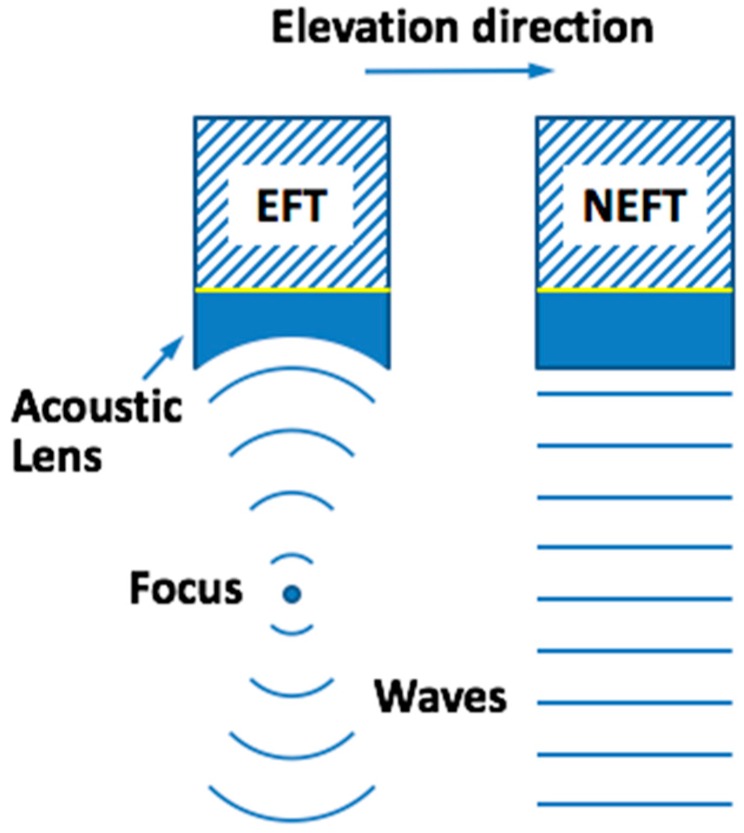
The difference between an elevation-focalized transducer (EFT) and a non-elevation-focalized transducer (NEFT) is that EFT has a constant self-focusing property in the elevation direction, but NEFT does not.

Transducer structure can greatly impact image quality through its remarkable influence on the distribution of an acoustic field. Conventional linear-array transducers demonstrate constant self-focusing properties in the elevation direction by changing the curvature of the acoustic lens before its piezoelectric material. Therefore, when doing PWUS, the ultrasound beam emitted by a traditional linear-array transducer is only “plane” in the azimuth direction. However, in the elevation direction there is a pre-defined physical focal depth, which means the beam is not “pure plane” in the 3-D space. This fact raises the question of whether, compared to a traditional elevation-focalized transducer (EFT), there will be a difference in image quality when doing PWUS with a non-elevation-focalized transducer (NEFT) (The difference between EFT and NEFT is shown in Figure 1). To answer this question, comparison was first made between the images obtained by the simulated NEFT and EFT transducers on two simulated phantoms using Matlab and Field II [15]. Then a NEFT probe was fabricated together with an EFT using the same parameters. Their acoustic fields in transmission were acquired using a 3-D acoustic field measurement system and examined. Finally, the images obtained with these two transducers on a standard ultrasound imaging phantom were compared.

## 2. Experimental Section

### 2.1. PWUS with Simulated Transducers

To make a guide and reference for the transducer fabrication and the imaging experiment set-up, an initial simulation study was conducted. PWUS images were obtained by the simulated NEFT and EFT transducers using Matlab (The Mathworks, Natick, MA, USA) and Field II [15], which is a widely-used ultrasound imaging simulation tool. The parameters used in the simulation are listed in Table 1. To simulate the effect of a self-focalized acoustic lens on EFT, the elements were mathematically divided into 10 equal parts in the elevation direction and given a fixed electric focus depth at 30 mm. For a fair comparison, the elements of NEFT were also divided into same-size segments in the elevation direction, but without performing an electric focusing.

**Figure 2:**
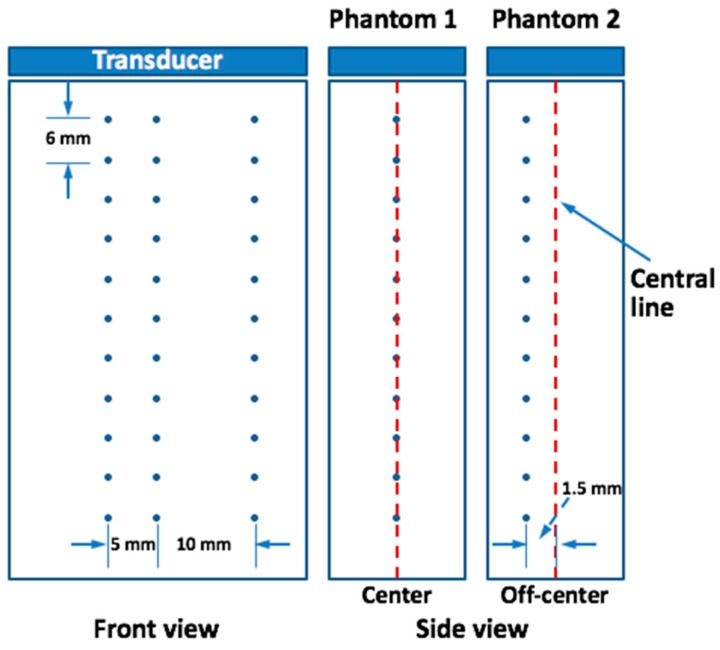
The front and side view of the simulated phantoms. In phantom 1, the elevation positions of the scatterers are in the center (*y* = 0 mm); but in phantom 2, the elevation positions of the scatterers are biased (*y* = 1.5 mm).

Two simulated phantoms were scanned without considering anisotropy. There were 30 point scatterers in each phantom, distributed within the depth range of *z =* 11 ~ 65 mm, with a 6 mm interval between the adjacent two rows. The lateral positions of the three scatterers in the same row were at *x =* −5 mm, 0 mm, and 10 mm. In the first phantom, the elevation positions of the scatterers were in the center (*y =* 0 mm), but in the second phantom, the elevation positions of the scatterers were biased (*y* = 1.5 mm). Sketches of the simulated phantoms from the front and side views are shown in Figure 2. The coherent compounding method [12] was used to obtain the image, with 11 angles synthesized with a 1-degree difference between each two adjacent angles. Multiple transmissions of the ultrasound plane-wave and corresponding reception of ultrasound echo signals were simulated with Field II. Then the obtained radio-frequency (RF) signals were reconstructed as B-mode images with a traditional delay-and-sum (DAS) beamforming method. Each group of 11 images obtained from different transmission angles were compounded into one image to enhance image quality. Finally, the images obtained by NEFT and EFT transducers and their profiles in the lateral direction at different depths were compared.

### 2.2. Evaluation of Acoustic Field

**Figure 3:**
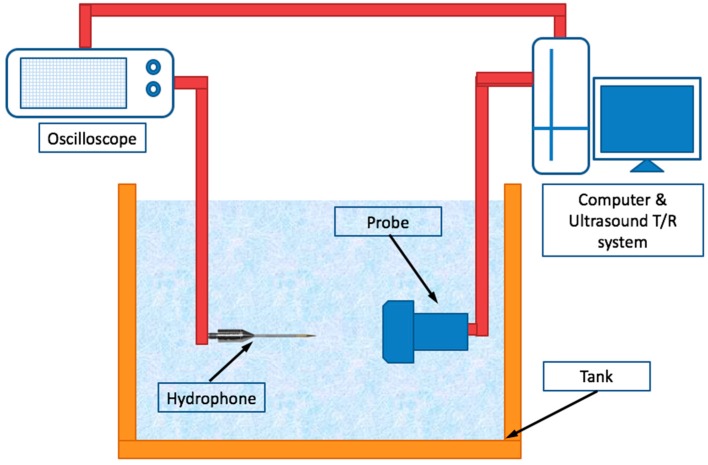
Diagram of the experimental setup for scanning the acoustic field in transmission of the fabricated EFT and NEFT transducers.

Both transducers used in the experiments described were 1-D linear array transducers with 128 elements. The EFT transducer (7.5-12840C, Shenzhen Shenchao Transducer, Shenzhen, China) has a fixed self-focus depth in the elevation direction at 30 mm. The NEFT transducer was made by the same manufacturer, and all its parameters were set to be the same as those of the EFT transducer, only without the elevation focus. The other parameters of these two transducers are listed in Table 1. A 3-D acoustic field measurement system (UMS3, Precision Acoustics, Dorchester, UK) with a needle-type hydrophone (SN2010, 0.5 mm diameter probe, Precision Acoustics, Dorchester, UK) was used to measure the acoustic characteristics of the two transducers in transmission. The transducers and the hydrophone were sunk into a tank which was filled with degassed water. By scanning the acoustic field with the moving hydrophone, the acoustic intensities at each scanning point on the X-Y plane were acquired as voltage signals in millivolt (mV) and shown on an oscilloscope. They were then transferred to a computer and plotted in a pseudo-color map with 100 × 100 points. Both transducers were driven by a programmable ultrasound research platform (V1, Verasonics, Kirkland, WA, USA), and the emission voltage was set to 30 V. Scanning was performed on the X-Y plane at different depths (*z* = 30 mm and *z* = 60 mm), and the step length of the hydrophone movement in X/Y direction was set to 0.5 mm. A diagram of the experimental setup is shown in Figure 3.

### 2.3. PWUS on a Standard Imaging Phantom

**Figure 4:**
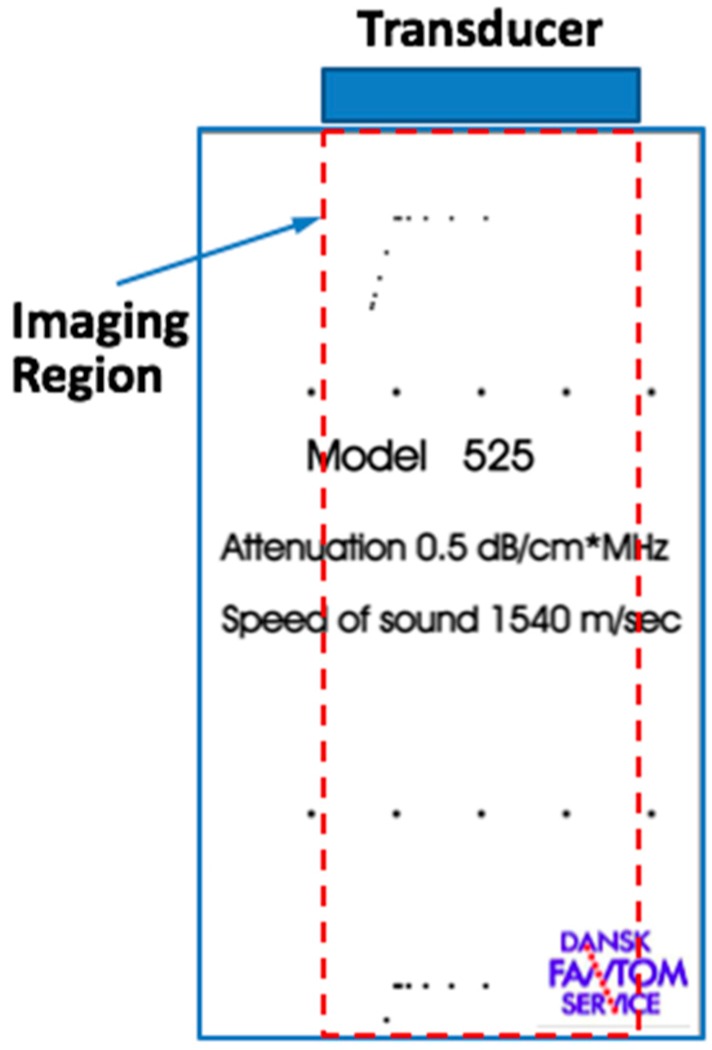
The front view of the standard imaging phantom (model 525, Dansk Fantom Service, Frederikssund, Denmark), and the imaging region used in the experiment of plane-wave ultrasound imaging (PWUS).

The imaging experiments were conducted with a commercial platform (V1, Verasonics, Kirkland, WA, USA) and a standard general-purpose ultrasound imaging phantom (model 525, Dansk Fantom Service, Denmark). A diagram of the experimental setup is shown in Figure 4. The point scatterers (nylon wires perpendicular to the X-Z plane) aligned in the horizontal direction are located at *z* = 11.6 mm, 34.0 mm and 89.3 mm depths. The parameters of the two transducers used for beamforming are listed in Table 1. The images obtained by NEFT and EFT transducers and their profiles in the lateral direction at different depths were compared.

## 3. Results and Discussion

### 3.1. Simulation Results

**Figure 5:**
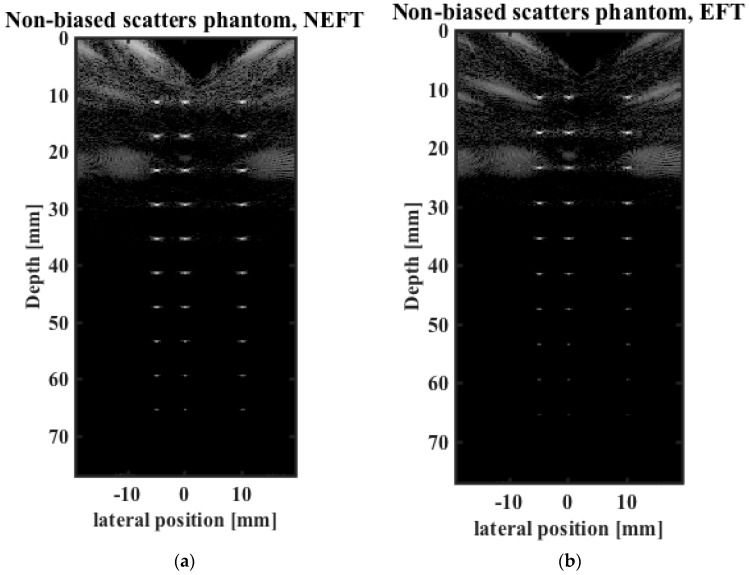
Images obtained using (**a**) NEFT; and (**b**) EFT on the “center” phantom which contains scatterers at the center of the elevation direction.

**Figure 6:**
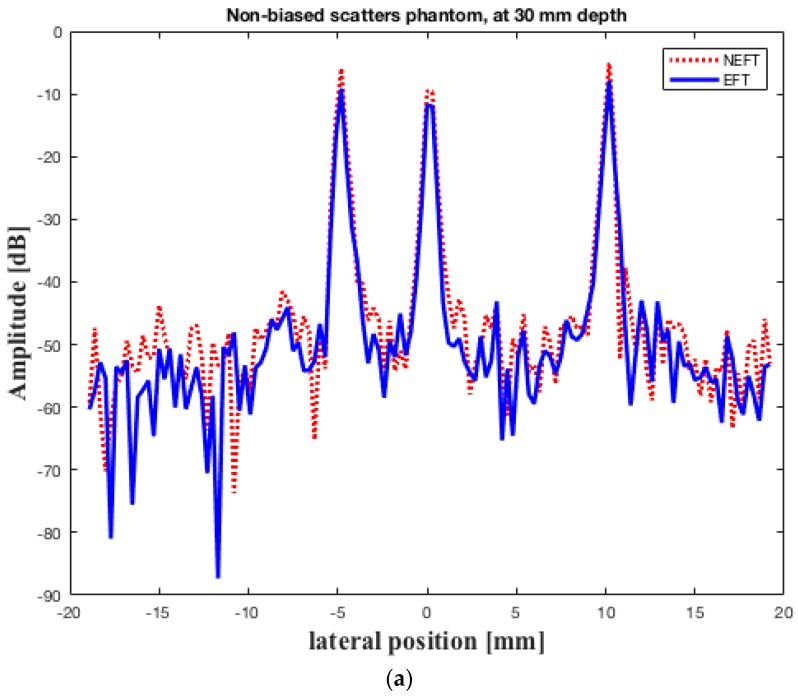

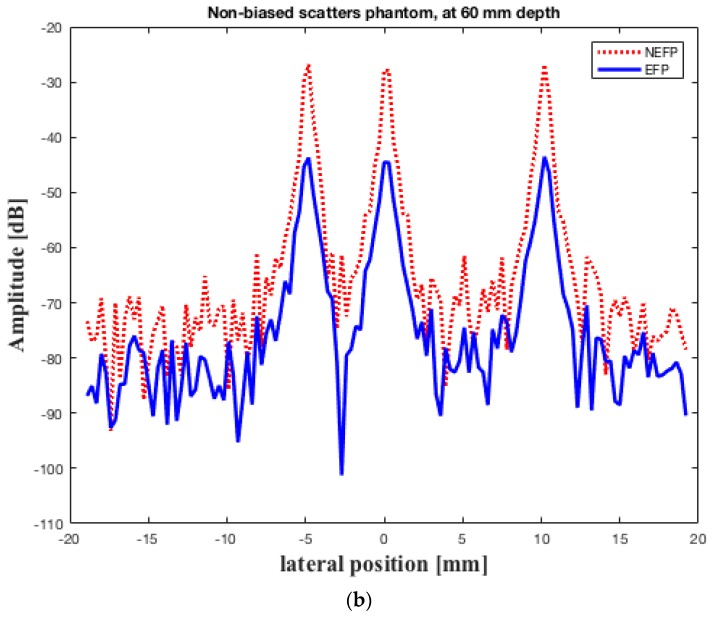
Lateral profiles of the image pixels obtained on the “center” phantom by the two transducers at (**a**) 30 mm depth; and (**b**) 60 mm depth. The constant focal depth in elevation direction of EFT is 30 mm. The 0 dB amplitude corresponds to the brightest pixel in the whole image.

**Figure 7:**
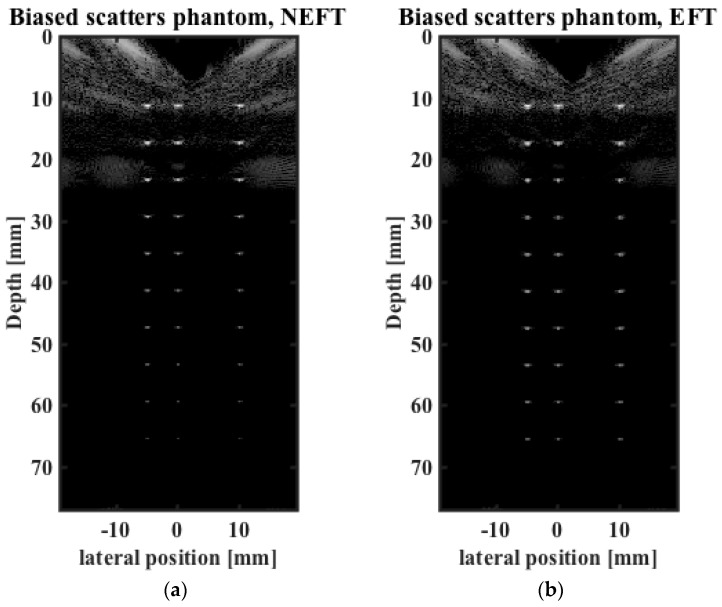
Images obtained using (**a**) NEFT; and (**b**) EFT on the “biased” phantom which contains scatterers at a biased position of the elevation direction.

**Figure 8:**
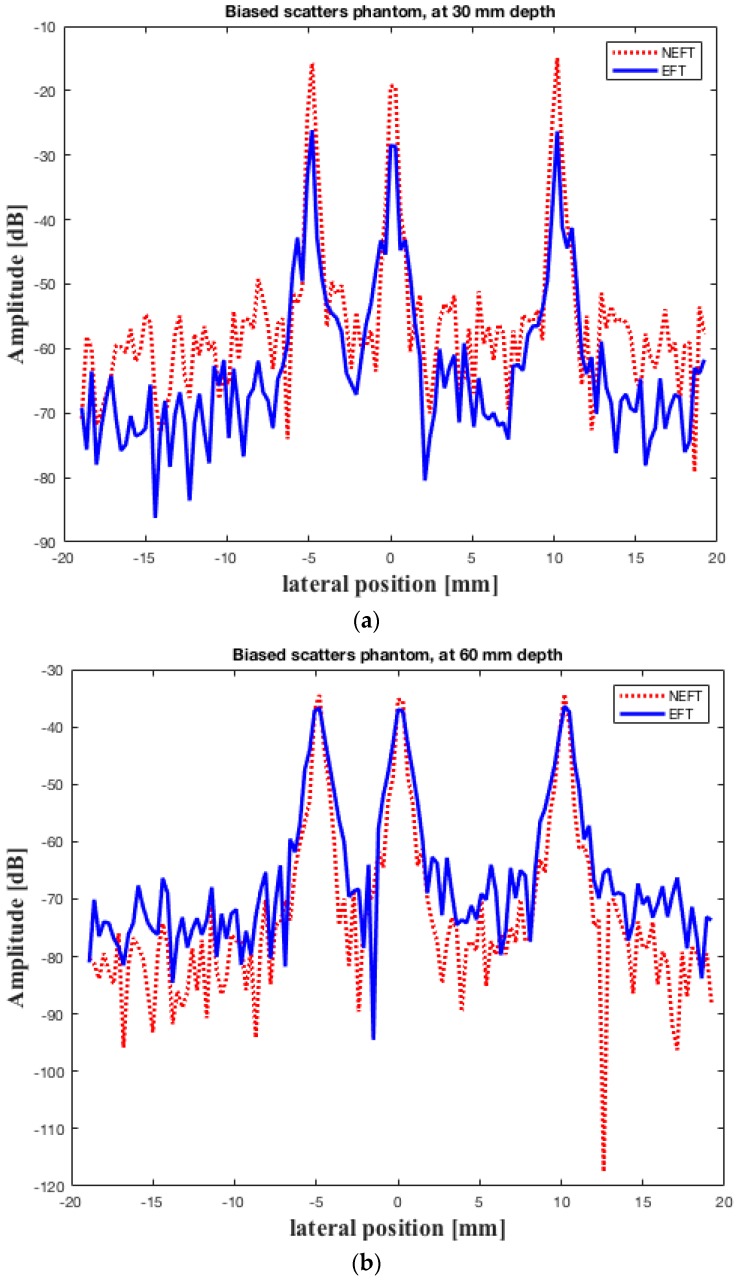
Lateral profiles of the image pixels obtained on the “biased” phantom by the two transducers at (**a**) 30 mm depth; and (**b**) 60 mm depth. The elevation focal depth of EFT is 30 mm. The 0 dB amplitude corresponds to the brightest pixel in the whole image.

The images obtained on the first simulated phantom (with scatterers at the center of the elevation direction, hereafter referred to as “center”) are shown in Figure 5. The lateral profiles of the image pixels at 30 mm depth, the constant focal depth in elevation direction of the EFT transducer, and at 60 mm depth are shown in Figure 6. The 0 dB amplitude corresponds to the brightest pixel in the whole image. The images obtained on the second simulated phantom (with scatterers biased in the elevation direction, hereafter referred to as “biased”) are shown in Figure 7. The lateral profiles at 30 mm and 60 mm depths are shown in Figure 8.

For the images obtained of the “center” phantom, considering the frequency-dependent attenuation, the brightness of the point scatterers should degrade uniformly with increasing depth, as shown in Figure 5a, which is obtained by NEFT. In comparison, in the image obtained by EFT (Figure 5b), the brightness degrades quickly with increasing depth, and a more distinct contrast is observed between the superficial and deep parts of the scan. In contrast, for the images obtained on the “biased” phantom, the brightness of the scatterers on the NEFT image degrades faster than that on the EFT image, as shown in Figure 7a,b. Moreover, the brightness on the EFT image is less uniform at different depths. As a consequence, when observer technician tries to aim the EFT transducer at the point scatterers, a mistake may be made if he/she thinks the brighter image (Figure 7b) is better than the darker one (Figure 5b). However, if the NEFT transducer is used, this mistake can be easily prevented, as the brighter image (Figure 5a) corresponds to the “center” phantom and the darker one (Figure 7a) corresponds to the “biased” phantom.

Figure 6 demonstrates that, for the “center” phantom, there seems to be no distinctive difference between the lateral resolution (which can be observed in the width of the peaks) and the image contrast (which can be observed in the amplitude difference between the peaks and the base line) of the images obtained using NEFT and EFT at 30 mm depth (Figure 6a). However, at 60 mm depth, the contrast of the NEFT image is somewhat better than the EFT image (Figure 6b). Similarly, for the “biased” phantom, the NEFT and EFT images exhibit no distinctive difference on the lateral resolution and contrast (Figure 8a). However, at 60 mm depth, the NEFT image shows a better lateral resolution than the EFT image (Figure 8b).

All these differences reflect the difference between the acoustic fields in transmission generated by the two transducers, as shown in the following section (Section 3.2), as the brightness of point scatterers is directly related to the acoustic energy trained on them, which also consequently influences the image resolution and contrast.

### 3.2. Scanning the Acoustic Field in Transmission

**Figure 9:**
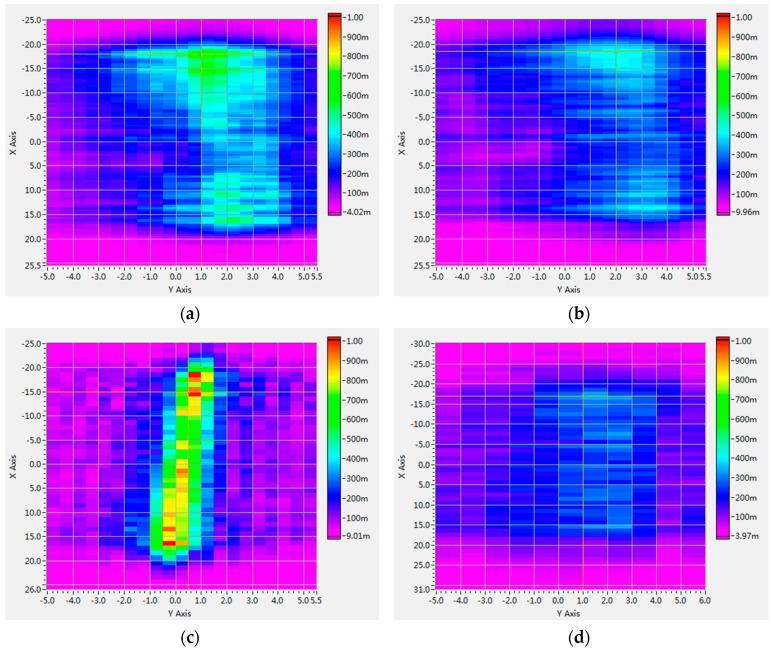
Acoustic intensity fields scanned on the X-Y planes of the NEFT at (**a**) 30 mm depth; and (**b**) 60 mm depth; and of the EFT at (**c**) 30 mm depth; and (**d**) 60 mm depth. The units of X/Y axis are both millimeters (mm). The pseudo color represents acoustic intensity values, which directly used the voltage signals output from the hydrophone (the unit is millivolt (mV)).

The acoustic intensity field scanned on the X-Y planes of the two transducers at different depths is shown in Figure 9. The units of the X/Y axis are both shown in millimeters (mm), and the pseudo color represents acoustic intensity values, which directly used voltage signals output from the hydrophone (the units are millivolts (mV)). Figure 9a,b shows the NEFT at 30 mm and 60 mm depths. At different depths, the acoustic intensity maps are both uniformly distributed, and there is no distinctive difference between their spreading patterns. Furthermore, at 60 mm depth, acoustic intensity is slightly lower than that at 30 mm depth. In contrast, for EFT at 30 mm depth, as shown in Figure 9c,d, the acoustic intensity distribution is much more concentrated than that of NEFT, due to the focusing effects of the acoustic lens. However, at 60 mm depth, the acoustic field becomes more dispersed, and acoustic intensity is also much lower than that of NEFT at the same depth.

### 3.3. PWUS with a Standard Imaging Phantom

**Figure 10:**
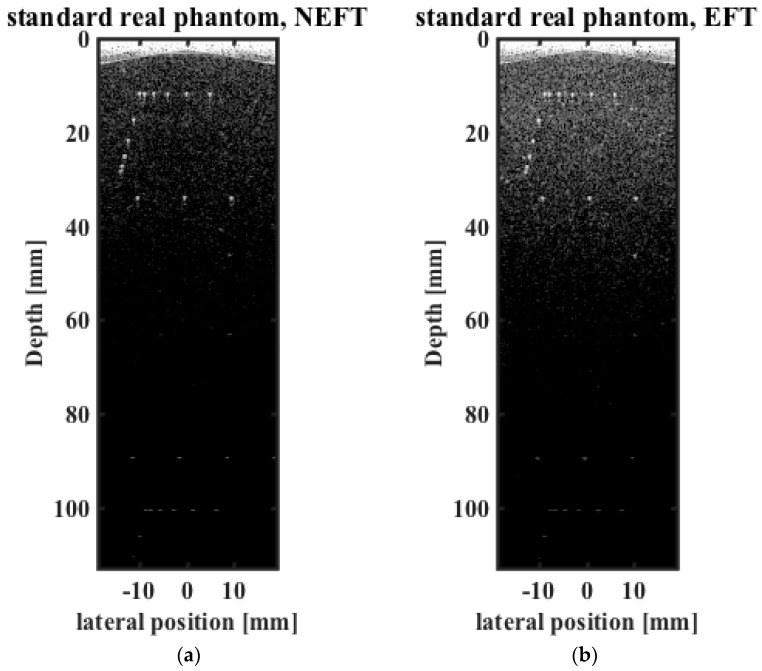
Images obtained on the standard ultrasound imaging phantom of point scatterers using (**a**) NEFT; and (**b**) EFT transducers.

**Figure 11:**
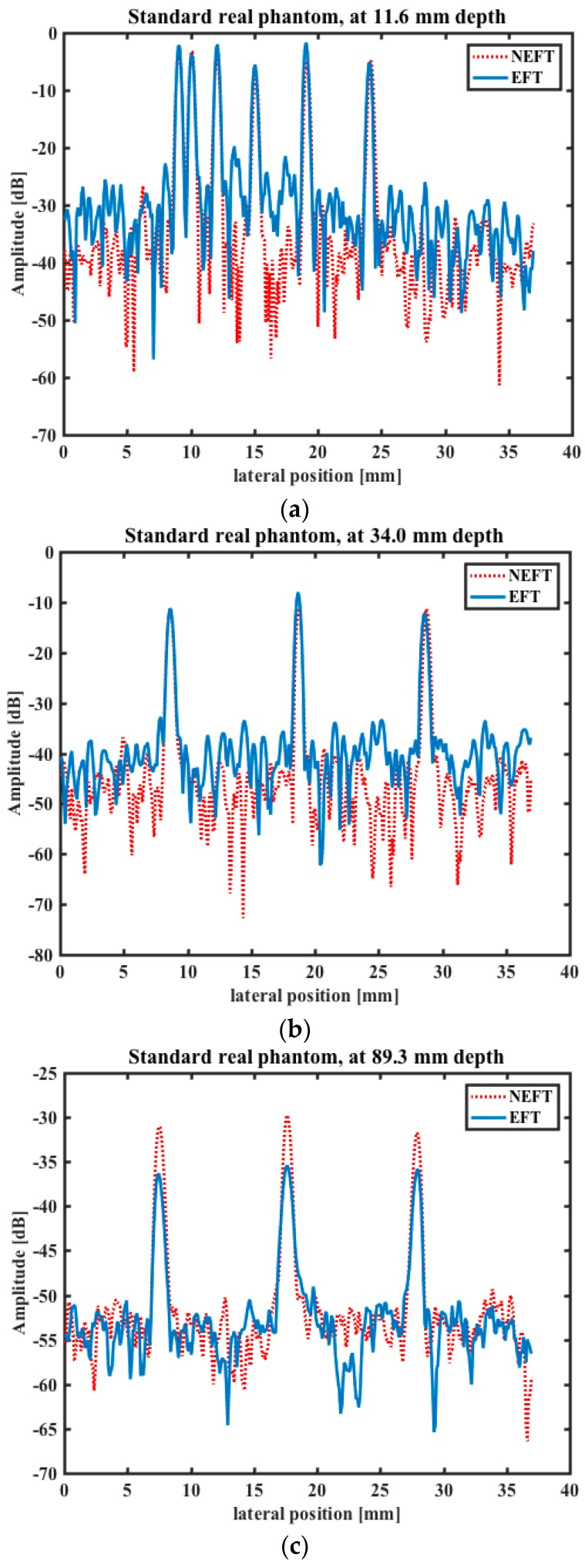
Lateral profiles of the image pixels obtained on the standard imaging phantom by the two transducers at (**a**) 11.6 mm; (**b**) 34.0 mm; and (**c**) 89.3 mm depths, where the point scatterers are located.

The imaging results of the phantom are shown in Figure 10 and Figure 11. It can be seen that in Figure 10, background brightness in the superficial region is reduced more by NEFT than by EFT. This phenomenon may be also related to the difference of the acoustic field intensity in transmission, as the imaging plane is at the center of the elevation direction (*y* = 0 mm), and the self-focusing of EFT causes more acoustic energy to be irradiated into this imaging plane and thus light up more in the background.

In Figure 11, the intensities of the image pixels at 11.6 mm, 34.0 mm and 89.3 mm depths are shown, as the point scatterers in this standard imaging phantom are located at these depths. Considering only the base line and disregarding the small fluctuation caused by the speckle pattern, at 11.6 mm and 34 mm depths the lateral resolution and image contrast of NEFT seem to be comparable to those of EFT. However, in the deeper region, i.e., at 89.3 mm depth, although lateral resolution is still similar, the image contrast of NEFT is better than that of EFT (about 5 dB higher). This can also be explained by the difference between their acoustic field in transmission. At those regions deeper than the elevational focal point, the acoustic energy irradiated by EFT is much more disperse than that of NEFT; thus, the amplitude of the echoes reflected from these regions will also be smaller than NEFT.

## 4. Conclusions

The brightness distribution in ultrasound images is not studied as much as other parameters, such as localized resolution and contrast, partially because it cannot be easily quantified. However, the difference in luminance between different regions or depths may mislead human vision in some ways, and may cause misdiagnosis in clinical practice. Ultrasound brightness is directly related to the amount of acoustic energy irradiated on the scatterers. In traditional ultrasound imaging, EFT is used, and the energy is concentrated on the focal area. In the X-Z plane, the focus can be adjusted by adjusting the transmission time on different elements of the transducer. However, in the Y-Z plane, the focus is fixed at a constant depth defined by the acoustic lens. This means that in traditional US imaging, the acoustic energy irradiated on the scatterers is not uniformly distributed and its pattern is complicated. However, in PWUS, all the elements of the transducer are driven simultaneously and an ultrasound plane-wave is transmitted, so that in the X-Z plane the acoustic energy irradiated into the object being scanned is nearly uniformly distributed, without respect to the effect of attenuation. However, if EFT is also used in the Y-Z plane, the distribution of acoustic energy will be controlled by the acoustic lens. Consequently, in the region near its constant focal depth, image brightness will be higher than the other regions. If the region of interest (ROI) is deeper than the focal depth, the human eye may give precedence to the brighter region at the focal depth, making the ROI harder to observed. However, if NEFT is used in PWUS, the acoustic field distribution in the X-Z and Y-Z planes will be nearly uniform. Thus disparities in the image brightness distribution will be minimized, and consequent distortion of the visual effect on the viewer will also be minimized.

To our knowledge, this study is the first to use the non-elevation-focalized transducer—which is never used in traditional ultrasound imaging—in PWUS and examine its effects. In this study, the image quality of PWUS obtained by both NEFT and EFT transducers was compared. The results indicate that, in regions deeper than the elevational focal point, the image contrast of NEFT is better than that of EFT. In addition, the study also demonstrates that, if an observer tries to aim the EFT transducer on point scatterers, a mistake may be made if he/she thinks the brighter image is better than the darker one. However, if the NEFT transducer is used, this mistake can be prevented.

To our knowledge, problems caused by the brightness distribution in PWUS images have attracted little attention, probably because the PWUS has not been widely used in clinical settings. Therefore, it is difficult to suggest suitable specific clinical applications for the PWUS using NEFT, especially for experienced ultrasound radiologists who have been accustomed to the images obtained by EFT using traditional methods. Studying clinical use and making such recommendations will be two of our next steps.

These results may not yet support particular applications in clinical practice, but, they can still inspire us to try to enhance the image quality of PWUS through a more sophisticated design of ultrasound transducer. In some cases, NEFT without any focusing effect may be not the best choice; selecting a deep constant focal depth in elevation direction and using the region between transducer and focal depth for imaging may be another area worthy of further study. Using a convex lens to broaden the ultrasound plane-wave in the elevation direction may also be worthwhile when trying to observe a large area through a small acoustic window. These assumptions will be further studied in the future.

## Figures and Tables

**Table 1:** Parameters of the simulated elevation-focalized transducer (EFT) and non-elevation-focalized transducer (NEFT), the manufactured EFT and NEFT transducers, and used for reconstructing the ultrasound images.

Parameters	Simulated EFT	Simulated NEFT	Manufactured EFT	Manufactured NEFT
Number of Elements	128	128	128	128
Center frequency (MHz)	7.5	7.5	7.5	7.5
Pitch (mm)	0.3	0.3	0.3	0.3
Height of element (mm)	5	5	5	5
Focus depth in elevation (mm)	30	--	30	--
Speed of sound (m/s)	1540	1540	1540	1540
Sampling frequency (MHz)	30	30	30	30
Dynamic range of image displaying (dB)	50	50	50	50
Frequency-dependent attenuation in (dB/[MHz·cm])	0.5	0.5	0.5	0.5
